# MRI-Negative Internuclear Ophthalmoplegia: A Diagnostic Challenge Emphasizing Clinical Localization

**DOI:** 10.7759/cureus.108492

**Published:** 2026-05-08

**Authors:** Moataz Mohamed, Abdulaziz Alharbi, Rahmah Alanazi, Fatima Alharbi

**Affiliations:** 1 General Medicine, Al Ribat National University, Khartoum, SDN; 2 General Surgery, King Salman Hospital, Riyadh, SAU; 3 Internal Medicine, King Salman Hospital, Riyadh, SAU; 4 Internal Medicine/Neurology, King Salman Hospital, Riyadh, SAU

**Keywords:** bilateral internuclear ophthalmoplegia, clinical localization, medial longitudinal fasciculus, mri-negative, multiple sclerosis

## Abstract

Internuclear ophthalmoplegia (INO) is a distinct ocular motility disorder localizing to the medial longitudinal fasciculus (MLF). Because the MLF is a microscopic, highly myelinated tract, small inflammatory lesions can cause significant functional conduction blocks that fall below the resolution threshold of typical 1.5T or 3T MRI. This leads to clinical radiographic dissociation, resulting in a significant diagnostic challenge when pathognomonic physical findings are present despite negative neuroimaging. A 16-year-old female presented with acute binocular diplopia and bilateral INO. Physical examination revealed pathognomonic bilateral failure of adduction, dissociated nystagmus, and impaired convergence. While the brain MRI demonstrated periventricular white matter hyperintensities, it failed to reveal a distinct lesion within the MLF. Despite the lack of definitive radiological localization in the brainstem, the clinical findings were conclusive for a demyelinating process. The patient was treated empirically with high-dose intravenous methylprednisolone to facilitate blood-brain barrier stabilization and address suspected inflammatory demyelination. This case underscores the diagnostic hierarchy in neurology, where pathognomonic physical findings must supersede nonspecific imaging in highly localizing brainstem disorders. Early recognition is vital, especially in pediatric populations where bilateral INO serves as a "red flag" for multiple sclerosis or other central demyelinating events.

## Introduction

Internuclear ophthalmoplegia (INO) is a unique ocular motility condition characterized by impaired adduction of the ipsilateral eye and dissociated nystagmus of the contralateral abducting eye during horizontal gaze [1]. The disorder results from dysfunction of the medial longitudinal fasciculus (MLF), a highly specialized, myelinated brainstem tract that coordinates conjugate eye movements by connecting the oculomotor nucleus in the midbrain with the abducens nucleus in the pons [2]. In pediatric and adolescent populations, bilateral INO is a particular clinical "red flag" for inflammatory demyelinating processes, most notably multiple sclerosis (MS). However, clinicians must also consider a broader differential in juveniles and adolescents, including brainstem gliomas, infectious rhombencephalitis, or rare metabolic disorders [3].

The 2017 revisions of the McDonald Criteria revolutionized the diagnosis of demyelinating illness by utilizing magnetic resonance imaging (MRI) to demonstrate dissemination in space and time [4]. Nevertheless, a substantial clinical challenge arises when pathognomonic neurological symptoms occur without conclusive radiographic data. Because the MLF is a microscopic structure, even modest inflammatory plaques can create symptomatic conduction blocks that fall below the resolution threshold of standard MRI sequences. If physicians prioritize imaging data over physical findings, this "radiographic-clinical dissociation" may delay necessary intervention. We describe the case of a 16-year-old girl with acute-onset bilateral INO and impaired convergence. This case highlights the importance of the clinical examination in the diagnostic hierarchy, demonstrating that a "negative" brainstem MRI should not preclude the prompt initiation of empirical therapy when clinical localization is definitive.

## Case presentation

A previously healthy 16-year-old female presented with a two-day history of binocular diplopia affecting both near and distant vision. The diplopia was initially associated with a mild headache but without progression in severity. She denied having a fever, recent respiratory infection, limb weakness, sensory disturbances, or imbalance. There was no prior history of neurological episodes or known autoimmune illness, and there was no relevant family history.

**Figure 1:**
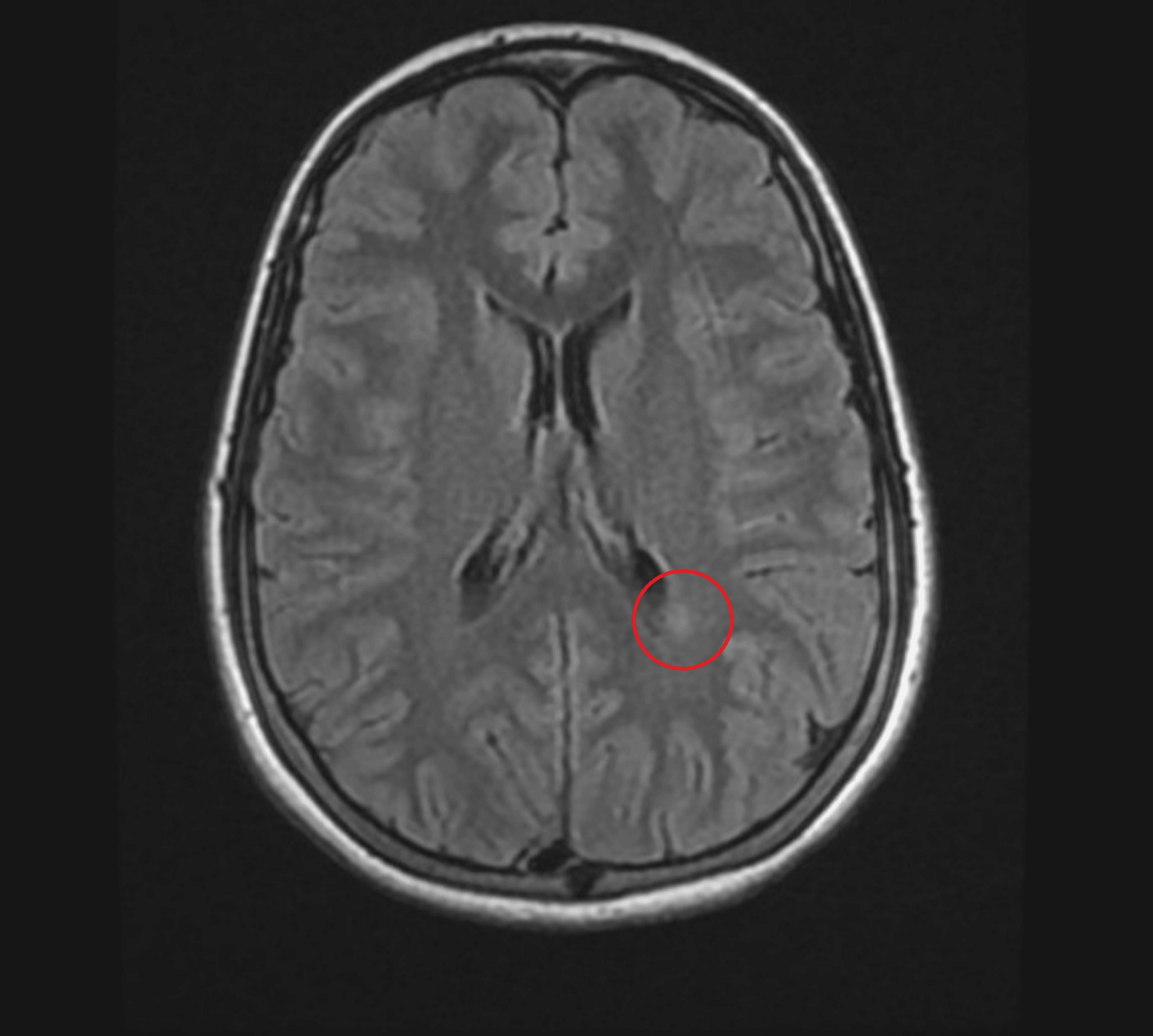
Cranial MRI findings (axial T2-FLAIR sequences) demonstrating a subtle posterior periventricular hyperintense lesion (indicated by the red circle). While the brainstem appeared radiographically normal, this periventricular finding in an adolescent patient serves as a critical indicator of a potential early demyelinating process, supporting the clinical suspicion of multiple sclerosis or clinically isolated syndrome. FLAIR: Fluid-Attenuated Inversion Recovery

**Figure 2:**
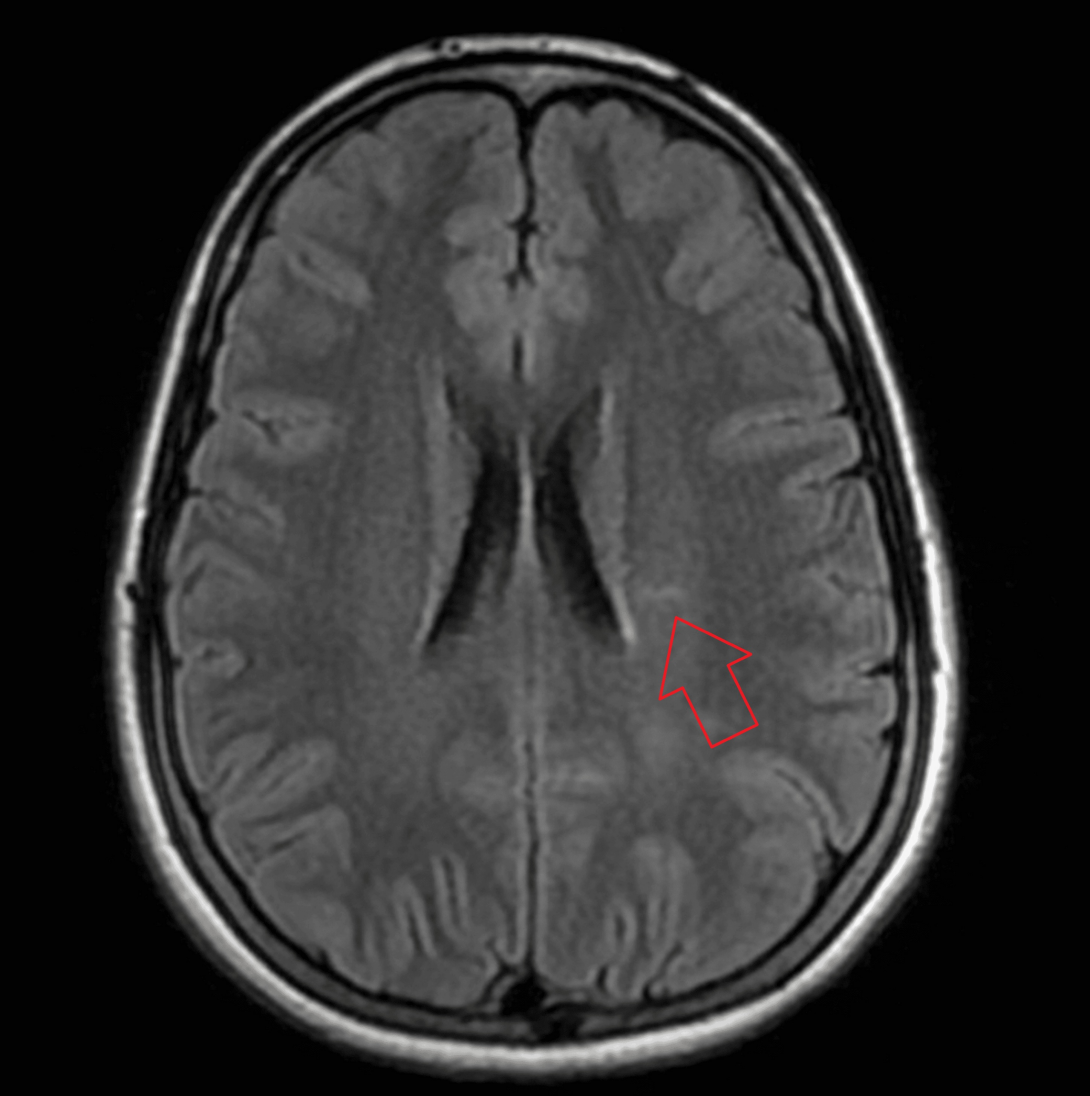
Cranial MRI findings (axial T2-FLAIR sequences) demonstrating a linear periventricular deep white matter hyperintensity perpendicular to the left lateral ventricle (indicated by the red arrow). This orientation is characteristic of "Dawson’s fingers," a radiographic hallmark of inflammatory demyelination resulting from perivenular inflammation. Its presence reinforces the clinical diagnosis of internuclear ophthalmoplegia as a demyelinating event despite the lack of visible lesions in the medial longitudinal fasciculus. FLAIR: Fluid-Attenuated Inversion Recovery

On examination, the patient was alert and hemodynamically stable. Cranial nerve examination revealed pathognomonic findings: bilateral limitation of adduction on horizontal gaze, accompanied by abducting nystagmus of the contralateral eye. Convergence was also impaired. These findings were diagnostic of bilateral INO. Motor examination demonstrated full strength in all four limbs, with normal tone and symmetrical reflexes. Sensory examination was intact to all modalities. Coordination testing and gait assessment were also noted to be normal, and no other focal neurological deficits were identified. MRI of the brain and spine with contrast demonstrated a linear periventricular deep white matter hyperintensity perpendicular to the body of the left lateral ventricle and two small posterior periventricular lesions (Figures 1, 2).

There was no diffusion restriction, no contrast enhancement, and no evidence of spinal cord lesions. The radiological impression favored nonspecific white matter changes. Magnetic resonance venography (MRV) was suboptimal but did not reveal any obvious abnormality. Laboratory investigations were largely unremarkable (Table 1), with the exception of a suppressed thyroid-stimulating hormone (TSH) level of 0.15 mIU/L. Follow-up thyroid function tests performed post-discharge showed normalization of TSH without intervention, suggesting the initial result was a transient physiologic stress response or non-thyroidal illness syndrome rather than primary thyroid pathology.

**Table 1:** Baseline Hematological, Biochemical, and Endocrine Laboratory Investigations. WBC: White Blood Cell; TSH: Thyroid-Stimulating Hormone; CRP: C-Reactive Protein; ESR: Erythrocyte Sedimentation Rate; HIV: Human Immunodeficiency Virus

Investigation	Result (Initial)	Result (Follow-Up)	Reference Range	Clinical Significance
WBC Count	7.22	15.21 (High)	4.2–10.8 × 10^9^/L	Leukocytosis (Secondary)
Neutrophils (%)	49.2	91.7 (High)	39–77%	Significant Neutrophilia
Lymphocytes (%)	41.6	7.4 (Low)	20–44%	Relative Lymphopenia
TSH	0.17 (L)	0.22 (Low)	0.652–4.409 mIU/L	Potential Hyperthyroidism
Free T4	-	15.61 / 17.01	13.4–21.1 pmol/L	Normal (Subclinical?)
Total Bilirubin	23 (H)	21 (High)	3.4–12.0 mmol/L	Isolated Hyperbilirubinemia
Potassium	3.91	3.7 (Low)	3.82–5.49 mmol/L	Mild Hypokalemia
CRP	-	<0.5 (Low)	0–10 mg/L	No Systemic Inflammation
ESR	-	6	0–15 mm/h	Normal
HIV 1 & 2	-	Non-Reactive	-	Negative Screen

Cerebrospinal fluid (CSF) analysis was sterile, with normal protein levels and no pleocytosis (Table 2), and was sent for a PCR panel, as shown in Table 3.

**Table 2:** CSF Analysis Including Cytology, Biochemistry, and Microbiology. CSF: Cerebrospinal Fluid; PCR: Polymerase Chain Reaction; WBC: White Blood Cell; RBC: Red Blood Cell

Investigation	Result	Reference Range	Interpretation
WBC Count	4	0–5 times 10^3^/µL	Normal (No Pleocytosis)
RBC Count	0	0 times 10^6^/µL	Non-traumatic Tap
Glucose	5.6 (High)	2.22–3.90 mmol/L	Hyperglycorrhachia
Protein	0.276	0.15–0.45 g/L	Normal
Gram Stain	Negative	No organisms	No acute infection
Culture	No Growth	No growth at 48h	Sterile CSF

**Table 3:** CSF PCR Analysis Results. CSF: Cerebrospinal Fluid; PCR: Polymerase Chain Reaction

Procedure	Result
*Neisseria meningitidis* (encapsulated)	Not Detected
Herpes simplex virus 1	Not Detected
Herpes simplex virus 2	Not Detected
Human herpesvirus 6	Not Detected
Enterovirus	Not Detected
Human parechovirus	Not Detected
Varicella-zoster virus	Not Detected
Cryptococcus gattii/Cryptococcus neoformans	Not Detected
Listeria monocytogenes	Not Detected
Streptococcus agalactiae	Not Detected
Streptococcus pneumoniae	Not Detected
Streptococcus pyogenes	Not Detected
Haemophilus influenzae	Not Detected
Mycoplasma pneumoniae	Not Detected

The autoimmune workup, including myelin oligodendrocyte glycoprotein (MOG-IgG) and aquaporin-4 (AQP4-IgG) antibodies, returned negative, effectively ruling out MOG-antibody disease (MOGAD) and neuromyelitis optica spectrum disorder (NMOSD). Given the patient’s age and the presence of bilateral INO, a demyelinating process was considered the most likely etiology. Early MS was strongly suspected despite the absence of definitive radiological criteria. An inflammatory or vasculitic brainstem process was also considered. A vascular etiology was deemed less likely given the patient’s age, absence of risk factors, and lack of diffusion restriction on imaging.

The patient was admitted and treated empirically with a five-day course of high-dose intravenous methylprednisolone. By the third day of therapy, she reported a significant subjective improvement in her diplopia, and a repeat neurological examination demonstrated a visible reduction in the adduction lag and nystagmus severity. A repeat neurological examination on the day of discharge demonstrated near-complete resolution of the adduction failure and a marked reduction in the amplitude of the abducting nystagmus.

At one-month follow-up, the patient remained clinically stable with no new neurological deficits. A follow-up brain MRI showed stable periventricular lesions with no evidence of new active inflammatory plaques, consistent with a clinically isolated syndrome (CIS). Long-term surveillance is ongoing to monitor for dissemination in time (DIT), as required for a definitive diagnosis of MS. 

## Discussion

Pathophysiological localization and the WEBINO (wall-eyed bilateral internuclear ophthalmoplegia) pattern

A hallmark sign of oculomotor dysconjugacy is the patient's sudden-onset binocular diplopia affecting both near and far vision. A lesion of the MLF was confirmed upon examination by the pathognomonic finding of bilateral adduction deficits. Specifically, there was a near-complete failure of adduction in both eyes during horizontal version, with the medial recti unable to bring either eye past the midline. This was accompanied by prominent, large-amplitude abducting nystagmus of the contralateral eye on attempted lateral gaze. Convergence was impaired, further localizing the pathology to the rostral midbrain [5]. The MLF is a heavily myelinated intersegmental tract that links the contralateral oculomotor nucleus in the midbrain and the abducens nucleus in the pons to coordinate conjugate horizontal gaze [6]. The internuclear fibers connecting the ipsilateral medial rectus subnucleus of CN III to the contralateral abducens nucleus are disrupted by a focal lesion in the MLF, typically in the dorsal pons or midbrain. In this state, the abducting eye may move outward frequently with nystagmus owing to an increased neural drive to overcome the failed conjugate response, although the abducens nucleus and lateral rectus continue to function [7]. This disorder is localized to the rostral midbrain, where the convergence centers reside, making this "WEBINO" pattern a crucial clinical point. Both medial recti fail in conjugate gaze due to the disruption of both internuclear pathways; furthermore, a tonic imbalance in the horizontal gaze network causes both eyes to deviate outward (exotropia). The WEBINO pattern typically indicates more widespread, midline, bilateral brainstem involvement than its isolated unilateral INO counterpart [8].

Radiographic-clinical dissociation and McDonald Criteria

The MRI-negative brainstem in this case, despite clear clinical localization, highlights the phenomenon of radiographic-clinical dissociation. The MLF is a minute structure, and inflammatory plaques large enough to cause symptomatic conduction blocks may fall below the resolution threshold of standard 1.5T or 3T MRI sequences; early demyelinating diseases are known to exhibit this radiographic-clinical contradiction [9,10]. According to the 2017 McDonald Criteria, the presence of periventricular lesions (Figures 1, 2) satisfies the requirement for Dissemination in Space (DIS), as these lesions are characteristic of a demyelinating process. However, because this was the patient's first neurological event and the imaging did not show simultaneous enhancing and non-enhancing lesions, the criteria for DIT were not yet met. Consequently, the presentation is best classified as a CIS.

Differential diagnosis and laboratory integration

The differential diagnosis prioritized inflammatory demyelination while systematically excluding mimics. Acute disseminated encephalomyelitis (ADEM) was considered less likely due to the absence of a prior upper respiratory tract infection or fever. Laboratory evaluation provided crucial information to rule out other comorbidities. Thyroid-related orbitopathy was initially considered due to the suppressed TSH (0.15 mIU/L) and normal free T4 (15.61 pmol/L) presented in the tables prior. However, rather than the particular adduction-nystagmus pattern of INO, thyroid orbitopathy usually manifests as a well-known manifestation of proptosis or restricted myopathy; however, an inadvertent subclinical autoimmune thyroid state, which is known to co-exist with other immune-mediated illnesses like MS, is most likely what this data indicates [11]. Regarding the initial endocrine findings, the suppressed TSH was discussed as a potential indicator of thyroid orbitopathy; however, its subsequent normalization post-discharge without intervention confirmed it as a transient physiologic stress response. This underscores the importance of interpreting isolated lab anomalies within the broader clinical context. The patient also experienced reactive neutrophilia (13.95 x 10^3^/µL) and leukocytosis (15.21 x 10^9^/L). Given the normal CRP (<0.5 mg/L) and ESR (6 mm/h), this was interpreted as a physiologic stress reaction to hospitalization rather than primary infection. With that being said, inflammatory demyelinating illnesses remain the priority for a 16-year-old female with bilateral brainstem symptoms. Although MS is the primary consideration, MOGAD and NMOSD have to be ruled out due to the lack of spinal cord lesions and contrast enhancement. A main vasculitic process or infectious rhombencephalitis was ruled out by the sterile CSF profile (WBC 4, normal protein). Moreover, there was no history of limb weakness, sensory abnormalities, or sphincter dysfunction, indicating that the inflammatory process and findings were physically limited to the midbrain and periventricular regions at the time of presentation.

Management rationale and clinical outcome

The management strategy involved a comprehensive initial workup, including a full neurological examination to assess for other brainstem, cerebellar, sensory, or pyramidal involvement. This was accompanied by a targeted MRI of the brain with thin-slice brainstem sequences and contrast to identify any potential MLF lesions, while prioritizing the exclusion of ischemia or masses. The diagnostic process also included screening for demyelinating etiologies, such as MOGAD (MOG-IgG) and NMOSD (AQP4-IgG), as well as infectious and systemic inflammatory conditions. Treatment for the acute episode focused on high-dose intravenous corticosteroid pulse therapy with methylprednisolone.

In the context of a suspected inflammatory demyelinating event, steroids serve to stabilize the blood-brain barrier by modulating tight junction proteins and suppressing pro-inflammatory cytokines, which helps restore functional conduction in the MLF [12,13]. Despite the nonspecific radiological appearance, the clinical severity of the bilateral INO justified the decision to treat empirically. Given the patient's lack of prior history of similar neurological events, the presentation was consistent with CIS.

Following the completion of the steroid pulse, the patient showed significant clinical improvement in ocular motility by the third day of treatment, with near-complete resolution by the time of discharge. Consequently, she was transitioned to a long-term management plan involving serial neuroimaging to monitor for further demyelinating events. The transition from acute hospital care to long-term neurological surveillance focused on patient education, ensuring that she understood the importance of follow-up imaging to monitor for characteristic DIT as part of her ongoing care. Serial neuroimaging remains necessary for a conclusive MS diagnosis. Finally, this case demonstrates the core principle that the clinical examination remains the most sensitive method for neuroanatomical localization, and a normal MRI does not rule out substantial brainstem disease.

## Conclusions

In conclusion, MRI is a vital tool in modern neurology, but it should be utilized in conjunction with careful and thorough history-taking and a comprehensive physical examination. This case demonstrates that in highly localizing brainstem disorders, clinical findings should guide acute management, even when standard neuroimaging is initially non-diagnostic. The patient’s significant clinical improvement following the initiation of high-dose corticosteroids supports the decision to prioritize pathognomonic physical signs over nonspecific imaging results.

However, it is crucial to acknowledge the limitations of this report, specifically the lack of long-term longitudinal data. While the initial response to empirical therapy was favorable, the definitive diagnosis of MS requires evidence of DIT, which can only be established through ongoing clinical and radiographic surveillance. Clinicians must maintain a high index of suspicion for "radiographic-clinical dissociation" in microscopic brainstem tracts and recognize that prompt intervention based on clinical sense is essential for managing acute neuro-inflammatory episodes.
